# Olfactory Sensitivity and Odor Structure-Activity Relationships for Aliphatic Carboxylic Acids in CD-1 Mice

**DOI:** 10.1371/journal.pone.0034301

**Published:** 2012-03-30

**Authors:** Selçuk Can Güven, Matthias Laska

**Affiliations:** Department of Physics, Chemistry and Biology, Linköping University, Linköping, Sweden; Barnard College, Columbia University, United States of America

## Abstract

Using a conditioning paradigm, the olfactory sensitivity of CD-1 mice for a homologous series of aliphatic n-carboxylic acids (ethanoic acid to n-octanoic acid) and several of their isomeric forms was investigated. With all 14 odorants, the animals significantly discriminated concentrations as low as 0.03 ppm (parts per million) from the solvent, and with four odorants the best-scoring animals even detected concentrations as low as 3 ppt (parts per trillion). Analysis of odor structure-activity relationships showed that the correlation between olfactory detection thresholds of the mice for the unbranched carboxylic acids and carbon chain length can best be described as a U-shaped function with the lowest threshold values at n-butanoic acid. A significant positive correlation between olfactory detection thresholds and carbon chain length of the carboxylic acids with their branching next to the functional carboxyl group was found. In contrast, no such correlation was found for carboxylic acids with their branching at the distal end of the carbon chain relative to the functional carboxyl group. Finally, a significant correlation was found between olfactory detection thresholds and the position of the branching of the carboxylic acids. Across-species comparisons suggest that mice are more sensitive for short-chained (C_2_ to C_4_) aliphatic n-carboxylic acids than other mammalian species, but not for longer-chained ones (C_5_ to C_8_). Further comparisons suggest that odor structure-activity relationships are both substance class- and species-specific.

## Introduction

The mouse is one of the most widely used animal models in olfactory research. Accordingly, the anatomy [1], [2], physiology [3], [4], and genetics of olfaction [5], [6], as well as the neural mechanisms underlying the coding of olfactory information [7], [8] have been studied intensively in this species. Only few studies, in contrast, have assessed olfactory sensitivity in the mouse at the organismal level [9], [10]. Such basic data of olfactory performance, however, are clearly important for the choice of adequate stimulus concentrations in electrophysiological or functional imaging studies of the olfactory system, or in studies assessing olfactory discrimination capabilities. Further, the assessment of olfactory detection thresholds for structurally related odorants allows us to elucidate possible correlations between molecular structural features and detectability of odor stimuli. Knowledge about such odor structure-activity relationships, in turn, gives us insight into receptor-ligand interactions and the neural coding of odor quality and intensity. A recent study, for example, has shown a significant positive correlation between the olfactory sensitivity of mice and the number of alkyl groups attached to a pyrazine ring [11]. Another study found that the combined presence or absence of two molecular structural features attached to a benzene ring may affect olfactory detection thresholds for aromatic aldehydes in the mouse by four orders of magnitude [12].

In the present study we have chosen aliphatic carboxylic acids as stimuli because of their behavioral relevance as important constituents of the mouse's vaginal secretion and general body odor [13], and because functional imaging studies have shown that they evoke distinguishably different odor maps in the mouse olfactory bulb which appear to correlate with certain molecular structural features of these odorants [7].

The possibility to obtain olfactory detection threshold values for both a homologous series of unbranched carboxylic acids as well as for some branched carboxylic acids allowed us to assess the impact of molecular structural features such as carbon chain length and presence/absence or position of branching of the carbon chain on detectability.

## Materials and Methods

### Ethics Statement

The experiments reported here comply with the *Guide for the Care and Use of Laboratory Animals* (National Institutes of Health Publication no. 86-23, revised 1985) and were performed according to a protocol approved by Linköping's Animal Care and Use Committee (Linköpings djurförsöksetiska nämnd, protocol #69-09).

### Animals

Testing was carried out using six male CD-1 mice (*Mus musculus*). The rationale for choosing this outbred strain of mice was to use animals with a genetic background that is more similar to wild-type mice than that of inbred strains. Furthermore, data on olfactory detection thresholds for a homologous series of aliphatic aldehydes [10], structurally related aromatic aldehydes [12], alkylpyrazines [11], monoterpenes [14], and amino acids [15] were obtained in earlier studies using the same mouse strain. Maintenance of the animals has been described in detail elsewhere [10]. The mice were 150–170 days old at the beginning of the study.

### Odorants

**Figure 1:**
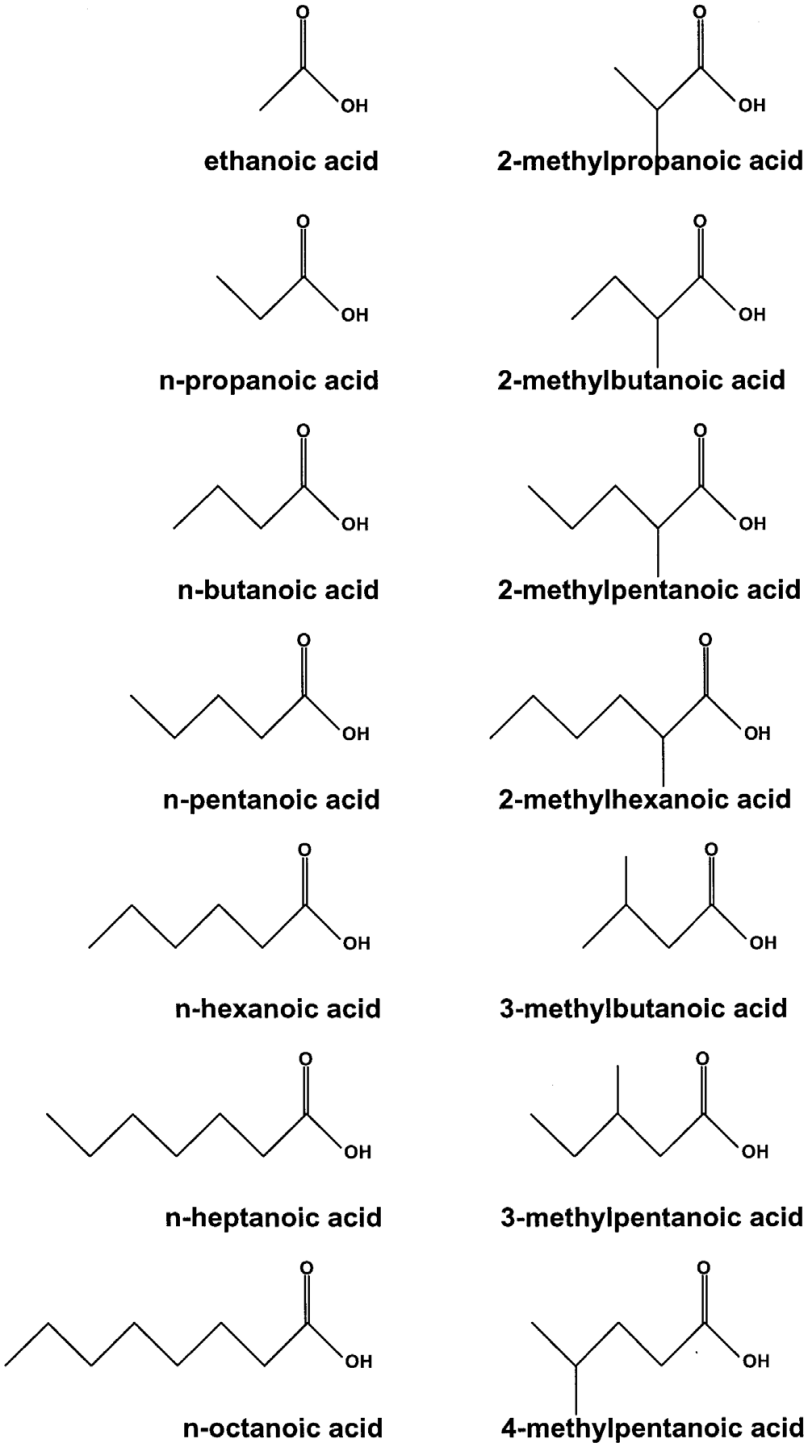
Chemical structure of the aliphatic carboxylic acids used.

A set of 14 odorants was used: ethanoic acid (CAS# 64-19-7), n-propanoic acid (CAS# 79-09-4), n-butanoic acid (CAS# 107-92-6), n-pentanoic acid (CAS# 109-52-4), n-hexanoic acid (CAS# 142-62-1), n-heptanoic acid (CAS# 111-14-8), n-octanoic acid (CAS# 124-07-2), 2-methylpropanoic acid (CAS# 79-31-2), 2-methylbutanoic acid (CAS# 116-53-0), 2-methylpentanoic acid (CAS# 97-61-0), 2-methylhexanoic acid (CAS# 4536-23-6), 3-methylbutanoic acid (CAS# 503-74-2), 3-methylpentanoic acid (CAS# 105-43-1), and 4-methylpentanoic acid (CAS# 646-07-1). The rationale for choosing these substances was to assesss the sensitivity of the mice for odorants representing members of a homologous series of aliphatic compounds, that is, substances sharing the same functional group but differing in carbon chain length. Additionally, we used isomeric forms of some of these compounds, that is, substances sharing the same sum formula and functional group but differing in branching of the carbon chain, allowing us to assess the impact of both structural features on detectability. All substances were obtained from Sigma-Aldrich (St. Louis, MO) and had a nominal purity of at least 99%. They were diluted using near-odorless diethyl phthalate (CAS# 84-66-2) as the solvent. Gas phase concentrations for the headspace above the diluted odorants were calculated using published vapor pressure data [16] and corresponding formulae [17]. Figure 1 shows the molecular structure of the odorants.

### Behavioral test

Olfactory sensitivity of the mice was assessed using an automated liquid-dilution olfactometer (Knosys, Tampa, FL) and an instrumental conditioning procedure which has been described in detail elsewhere [18]. Briefly, animals were trained to insert their snout into the odor sampling port of a test chamber. This triggered a 2 s presentation of either an odorant used as the rewarded stimulus (S+) or a blank (headspace of the solvent) used as the unrewarded stimulus (S−). Licking at a steel tube providing 2.5 µl of water reinforcement in response to presentation of the S+ served as the operant response. Forty such trials (20 S+ and 20 S− trials in pseudorandomized order) using the same concentration of a given S+ were conducted per animal and condition.

Olfactory detection thresholds were determined by testing the animals' ability to discriminate between increasing dilutions of an odorant used as S+, and the solvent alone used as S−. Starting with a gas phase concentration of 1 ppm, each stimulus was successively presented in 10-fold dilution steps until an animal failed to significantly discriminate the odorant from the solvent. Subsequently, an intermediate concentration (0.5 log units between the lowest concentration that was detected above chance and the first concentration that was not) was tested in order to determine the threshold value more exactly.

The 14 odorants were tested with all six animals in the following order: n-butanoic acid, n-octanoic acid, n-propanoic acid, n-heptanoic acid, ethanoic acid, n-hexanoic acid, n-pentanoic acid, 2-methylpentanoic acid, 2-methylpropanoic acid, 3-methylpentanoic acid, 2-methylhexanoic acid, 2-methylbutanoic acid, 3-methylbutanoic acid, 4-methylpentanoic acid.

### Data analysis

For each individual animal, the percentage of correct choices from 40 consecutive trials per dilution step was calculated. Correct choices consisted both of licking in response to presentation of the S+ and not licking in response to the S−, and errors consisted of animals showing the reverse pattern of operant responses, that is: not licking in response to the S+ and licking in response to the S−. Post-hoc analyses showed that errors almost exclusively consisted of false positives (that is, licking in response to presentation of the S−) when clearly detectable concentrations were presented. At perithreshold concentrations, in contrast, errors were more evenly distributed between false positives and false negatives (that is, not licking in response to the S+). Significance levels were determined by calculating binomial z-scores corrected for continuity from the number of correct and false responses for each individual and condition. All tests were two-tailed and the alpha level was set at 0.01.

Correlations between olfactory threshold values and molecular parameters such as carbon chain length of the odorants tested or position of branching of the carbon chain were calculated using linear regression analysis and, in the case of the unbranched carboxylic acids, also using third-order polynomial regression analysis. Within-species comparisons of performance were performed using the Wilcoxon signed rank test for dependent samples.

## Results

### Olfactory sensitivity

**Figure 2:**
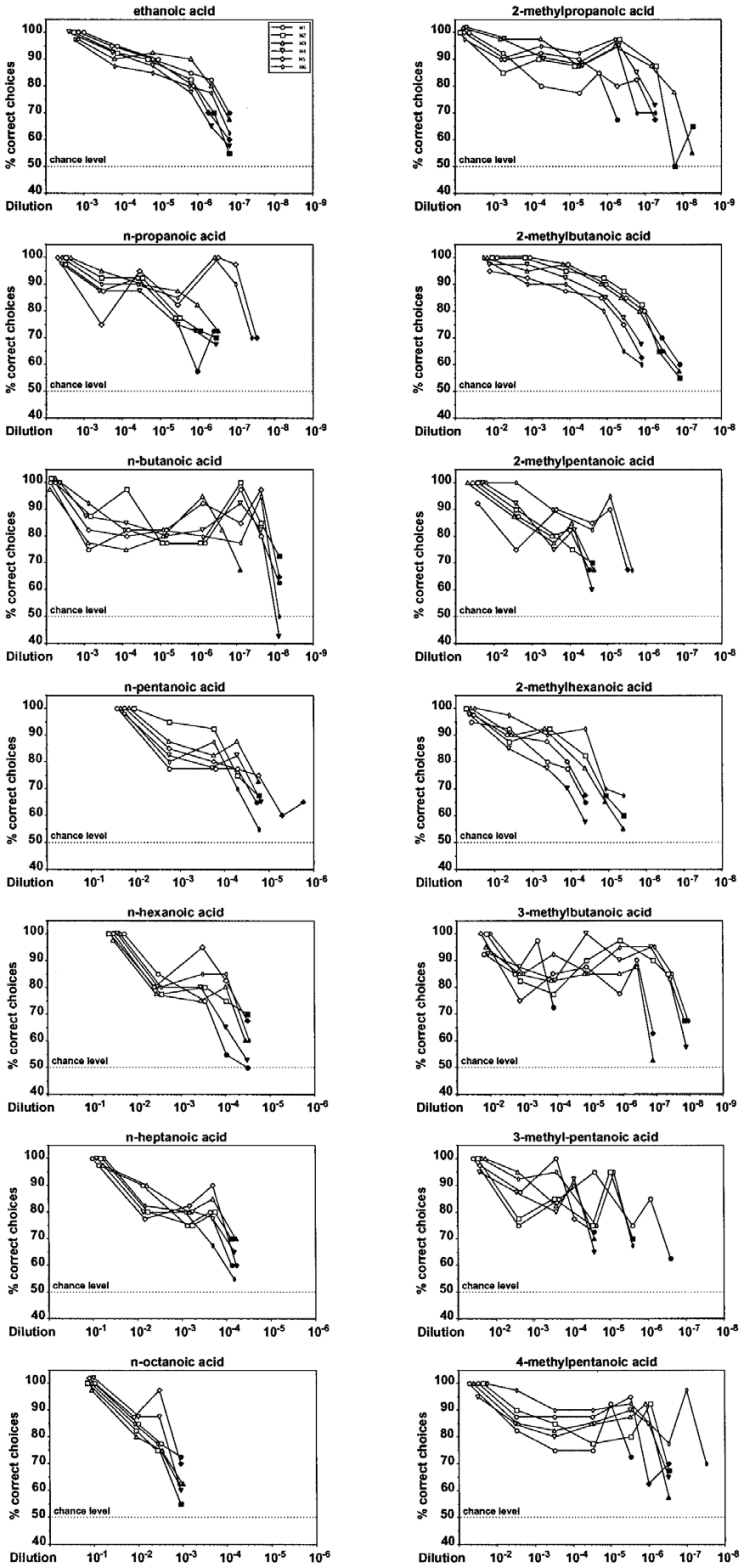
Performance of CD-1 mice in discriminating between various dilutions of an aliphatic carboxylic acid and the solvent. Each data point represents the percentage of correct choices from a total of 40 decisions per individual animal. The six different symbols represent data from each of the six individual animals tested per odorant. Filled symbols indicate dilutions that were not discriminated significantly above chance level (binomial test, p>0.01).

Figure 2 shows the performance of the mice in discriminating between various dilutions of a given odorant and the solvent. All six animals significantly distinguished dilutions as low as 1∶650,000 ethanoic acid, 1∶290,000 n-propanoic acid, 1∶4,333,000 n-butanoic acid, 1∶5,900 n-pentanoic acid, 1∶3,100 n-hexanoic acid, 1∶1,500 n-heptanoic acid, 1∶300 n-octanoic acid, 1∶600,000 2-methylpropanoic acid, 1∶79,000 2-methybutanoic acid, 1∶11,700 2-methylpentanoic acid, 1∶2,500 2-methylhexanoic acid, 1∶2,633 3-methylbutanoic acid, 1∶11,700 3-methylpentanoic acid, and 1∶100,000 4-methylpentanoic acid from the solvent (binomial test, p<0.01), with some individuals even scoring better. (Please note that the headspace above these dilutions was further diluted by a factor of 40 by the olfactometer used with the mice.)

The individual mice generally demonstrated similar detection threshold values with a given odorant and with eight of the 14 odorants they differed only by a dilution factor of 10 (n-butanoic acid, n-pentanoic acid, 2-methylbutanoic acid, 2-methylpentanoic acid, 2-methylhexanoic acid) or a factor of 3 (ethanoic acid, n-hexanoic acid, n-heptanoic acid) between the highest- and the lowest-scoring animal. In the case of n-octanoic acid, all six animals even displayed the same detection threshold value. With one odorant (n-propanoic acid) the range of threshold values was a factor 33, and with three odorants (2-methylpropanoic acid, 3-methylpentanoic acid, 4-methylpentanoic acid) it was a factor of 100. The largest difference in sensitivity for a given odorant between individuals was a dilution factor of 10,000 and was found with 3-methylbutanoic acid for which one individual mouse was three orders of magnitude less sensitive than the other five animals.

Table 1 summarizes the threshold dilutions of the mice and shows various measures of corresponding gas phase concentrations [17] allowing readers to easily compare the data obtained in the present study to those reported by other authors using one of these convertible measures. In all cases, threshold dilutions correspond to gas phase concentrations ≤0.03 ppm (parts per million). With four odorants (n-butanoic acid, 2-methylpropanoic acid, 3-methylbutanoic acid, 4-methylpentanoic acid) individual animals even reached threshold values as low as 3 ppt (parts per trillion).

**Table 1:** Olfactory detection threshold values in CD-1 mice for aliphatic carboxylic acids, expressed in various measures of gas phase concentrations.

		liquid	gas phase concentration				
odorant	n	dilution	molec./cm^3^	ppm	log ppm	Mol/l	log Mol/l
ethanoic acid	3	1∶650,000	2.5•10^10^	0.001	−3.00	4.5•10^−11^	−10.35
	3	1∶2,166,667	7.5•10^9^	0.0003	−3.52	1.3•10^−11^	−10.87
n-propanoic acid	3	1∶290,000	2.5•10^10^	0.001	−3.00	4.5•10^−11^	−10.35
	1	1∶966,667	7.5•10^9^	0.0003	−3.52	1.3•10^−11^	−10.87
	2	1∶9,667,000	7.5•10^8^	0.00003	−4.52	1.3•10^−12^	−11.87
n-butanoic acid	1	1∶4,333,000	7.5•10^8^	0.00003	−4.52	1.3•10^−12^	−11.87
	5	1∶43,333,000	7.5•10^7^	0.000003	−5.52	1.3•10^−13^	−12.87
n-pentanoic acid	1	1∶5,900	2.5•10^11^	0.01	−2.00	4.5•10^−10^	−9.35
	4	1∶19,667	7.5•10^10^	0.003	−2.52	1.3•10^−10^	−9.87
	1	1∶59,000	2.5•10^10^	0.001	−3.00	4.5•10^−11^	−10.35
n-hexanoic acid	2	1∶3,100	2.5•10^11^	0.01	−2.00	4.5•10^−10^	−9.35
	4	1∶10,333	7.5•10^10^	0.003	−2.52	1.3•10^−10^	−9.87
n-heptanoic acid	1	1∶1,500	2.5•10^11^	0.01	−2.00	4.5•10^−10^	−9.35
	5	1∶5,000	7.5•10^10^	0.003	−2.52	1.3•10^−10^	−9.87
n-octanoic acid	6	1∶300	7.5•10^11^	0.03	−1.52	1.3•10^−9^	−8.87
2-methyl-	1	1∶600,000	7.5•10^9^	0.0003	−3.52	1.3•10^−11^	−10.87
propanoic acid	1	1∶1,800,000	2.5•10^9^	0.0001	−4.00	4.5•10^−12^	−11.35
	2	1∶6,000,000	7.5•10^8^	0.00003	−4.52	1.3•10^−12^	−11.87
	1	1∶18,000,000	2.5•10^8^	0.00001	−5.00	4.5•10^−13^	−12.35
	1	1;60,000,000	7.5•10^7^	0.000003	−5.52	1.3•10^−13^	−12.87
2-methyl-	1	1∶79,000	2.5•10^10^	0.001	−3.00	4.5•10^−11^	−10.35
butanoic acid	2	1∶263,333	7.5•10^9^	0.0003	−3.52	1.3•10^−11^	−10.87
	3	1∶790,000	2.5•10^9^	0.0001	−4.00	4.5•10^−12^	−11.35
2-methyl-	4	1∶11,700	7.5•10^10^	0.003	−2.52	1.3•10^−10^	−9.87
pentanoic acid	2	1∶117,000	7.5•10^9^	0.0003	−3.52	1.3•10^−11^	−10.87
2-methyl-	1	1∶2,500	2.5•10^11^	0.01	−2.00	4.5•10^−10^	−9.35
hexanoic acid	2	1∶8,333	7.5•10^10^	0.003	−2.52	1.3•10^−10^	−9.87
	3	1∶25,000	2.5•10^10^	0.001	−3.00	4.5•10^−11^	−10.35
3-methyl-	1	1∶2,633	7.5•10^11^	0.03	−1.52	1.3•10^−9^	−8.87
butanoic acid	2	1∶2,633,333	7.5•10^8^	0.00003	−4.52	1.3•10^−12^	−11.87
	3	1∶26,333,333	7.5•10^7^	0.000003	−5.52	1.3•10^−13^	−12.87
3-methyl-	3	1∶11,700	7.5•10^10^	0.003	−2.52	1.3•10^−10^	−9.87
pentanoic acid	2	1∶117,000	7.5•10^9^	0.0003	−3.52	1.3•10^−11^	−10.87
	1	1;1,170,000	7.5•10^8^	0.00003	−4.52	1.3•10^−12^	−11.87
4-methyl-	1	1∶100,000	7.5•10^9^	0.0003	−3.52	1.3•10^−11^	−10.87
pentanoic acid	1	1∶330,000	2.5•10^9^	0.0001	−4.00	4.5•10^−12^	−11.35
	3	1∶1,000,000	7.5•10^8^	0.00003	−4.52	1.3•10^−12^	−11.87
	1	1;10,000,000	7.5•10^7^	0.000003	−5.52	1.3•10^−13^	−12.87

n indicates the number of animals.

### Odor structure-activity relationships

**Figure 3:**
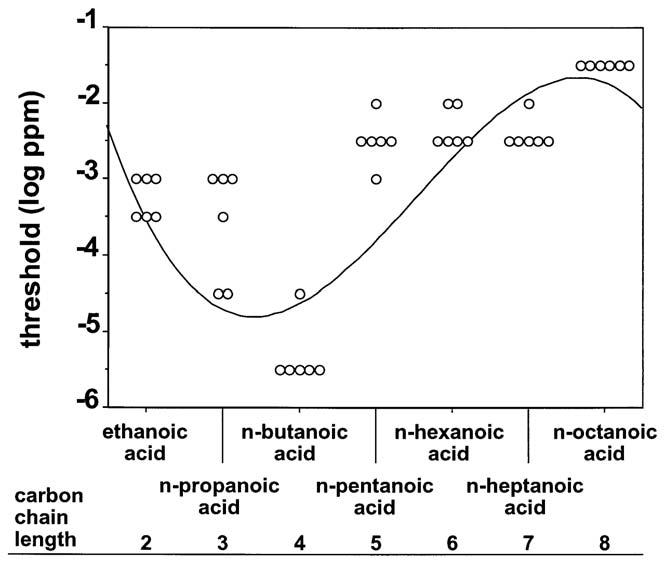
Olfactory detection thresholds of CD-1 mice for the seven aliphatic carboxylic acids with an unbranched carbon backbone tested. Each symbol represents the threshold value of an individual animal. The solid line indicates the regression with the best goodness-of-fit according to third order polynomial regression analysis (R^2^ = 0.60, p<0.001, equation for line of best fit: y = 3.476−5.288x+1.117x^2^−0.067x^3^).

Figure 3 shows the olfactory detection threshold values of the mice for the seven aliphatic carboxylic acids with an unbranched carbon chain tested here. Thresholds decreased from ethanoic acid to n-butanoic acid, followed by an increase in thresholds from n-butanoic acid to n-octanoic acid. Accordingly, linear regression analysis found a significant negative slope for olfactory detection thresholds and carbon chain lengths C_2_ to C_4_ (R^2^ = 0.69, p<0.001, equation for line of best fit: y = −1.04x−0.93), and a significant positive slope for carbon chain lengths C_4_ to C_8_ (R^2^ = 0.68, p<0.001, equation for line of best fit: y = 0.77x−7.47). Thus, the correlation between olfactory detection thresholds of the mice for the unbranched carboxylic acids and carbon chain lengths C_2_ to C_8_ can best be described as a U-shaped function (third order polynomial regression, R^2^ = 0.60, p<0.001, equation for line of best fit: y = 3.476−5.288x+1.117x^2^−0.067x^3^).

**Figure 4:**
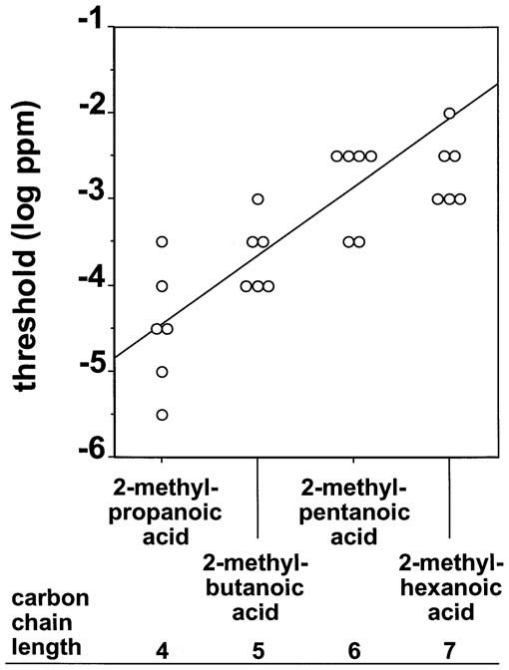
Olfactory detection thresholds of CD-1 mice for the four aliphatic carboxylic acids with the branching of the carbon chain next to the functional carboxyl group. Each symbol represents the threshold value of an individual animal. The solid line indicates the regression with the best goodness-of-fit according to linear regression analysis (R^2^ = 0.66, p<0.001, equation for line of best fit: y = 0.63x−6.90).

Figure 4 shows the olfactory detection threshold values of the mice for the four aliphatic carboxylic acids with the branching of the carbon chain next to the functional carboxyl group. Thresholds increased from 2-methylpropanoic acid to 2-methylhexanoic acid. Accordingly, linear regression analysis found a significant positive slope for olfactory detection thresholds and carbon chain lengths C_4_ to C_7_ of the carboxylic acids with their branching next to the functional carboxyl group (R^2^ = 0.66, p<0.001, equation for line of best fit: y = 0.63x−6.90).

**Figure 5:**
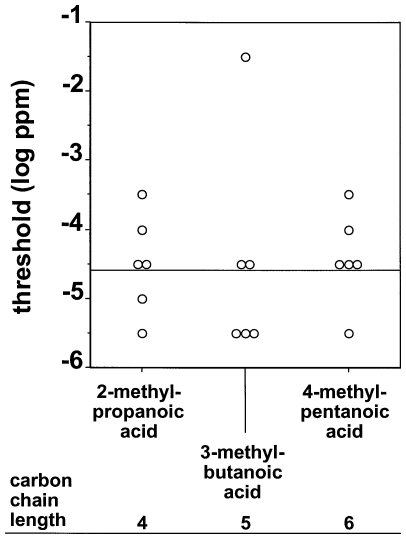
Olfactory detection thresholds of CD-1 mice for the three aliphatic carboxylic acids with the branching at the distal end of the carbon chain relative to the functional carboxyl group. Each symbol represents the threshold value of an individual animal. The solid line indicates the regression with the best goodness-of-fit according to linear regression analysis (R^2^ = 0.0016, p>0.05, equation for line of best fit: y = 0.04x−4.68).

Figure 5 shows the olfactory detection threshold values of the mice for the three aliphatic carboxylic acids with the branching at the distal end of the carbon chain relative to the functional carboxyl group. Thresholds did not systematically vary between 2-methylpropanoic acid, 3-methylbutanoic acid, and 4-methylpentanoic acid. Accordingly, linear regression analysis did not find a significant slope for olfactory detection thresholds and carbon chain lengths C_4_ to C_6_ of the carboxylic acids with their branching at the distal end of the carbon chain relative to the functional carboxyl group (R^2^ = 0.0016, p>0.05, equation for line of best fit: y = 0.04x−4.68).

**Figure 6:**
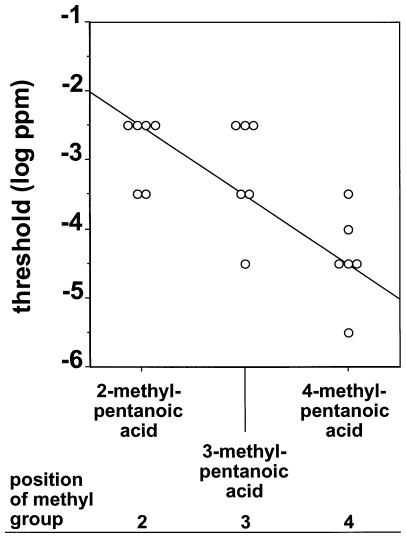
Olfactory detection thresholds of CD-1 mice for the three aliphatic carboxylic acids with six carbons and a branching of the carbon chain. Each symbol represents the threshold value of an individual animal. The solid line indicates the regression with the best goodness-of-fit according to linear regression analysis (R^2^ = 0.49, p<0.001, equation for line of best fit: y = −0.79x−1.10).

Figure 6 shows the olfactory detection threshold values of the mice for the three aliphatic carboxylic acids with six carbons and a branching of the carbon chain. Thresholds decreased from 2-methylpentanoic acid over 3-methylpentanoic acid to 4-methylpentanoic acid. Accordingly, linear regression analysis found a significant negative slope for olfactory detection thresholds and and position of the branching of the C_6_ carboxylic acids (R^2^ = 0.49, p<0.001, equation for line of best fit: y = −0.79x−1.10).

No significant difference was found between the olfactory detection thresholds of the unbranched carboxylic acids with a carbon chain length C_4_ to C_7_ (n-butanoic acid to n-heptanoic acid) and carboxylic acids with the same number of carbons and their branching next to the functional carboxyl group (2-methylpropanoic acid to 2-methylhexanoic acid) (Wilcoxon, p>0.05). In contrast, a significant difference was found between the olfactory detection thresholds of the unbranched carboxylic acids with a carbon chain length C_4_ to C_6_ (n-butanoic acid to n-hexanoic acid) and carboxylic acids with the same number of carbons and their branching at the distal end of the carbon chain (2-methylpropanoic acid to 4-methylpentanoic acid) (Wilcoxon, p<0.01). Here, the mice showed a significantly higher sensitivity, that is, lower detection thresholds for the iso-forms of the carboxylic acids than for the n-forms.

## Discussion

The results of the present study demonstrate that mice have a well-developed olfactory sensitivity for monomolecular odorants belonging to the chemical class of aliphatic carboxylic acids. Further, they demonstrate significant correlations between olfactory detection thresholds and molecular structural features of the carboxylic acids such as carbon chain length and presence or position of branching of the carbon chain.

### Olfactory sensitivity

Although only six mice were tested per stimulus, the results appear robust as interindividual variability was generally low and considerably smaller than the range reported in studies on human olfactory sensitivity, that is, within three orders of magnitude [19]. In fact, with the majority of odorants the largest difference between the highest- and the lowest-scoring animal with a given stimulus was a factor of 10 or lower (see Fig. 2). Further, for all odorants, the animals' performance with the lowest concentrations presented dropped to chance level, suggesting that the statistically significant discrimination between higher concentrations of a stimulus and the solvent was indeed based on chemosensory perception and not on other cues.

**Figure 7:**
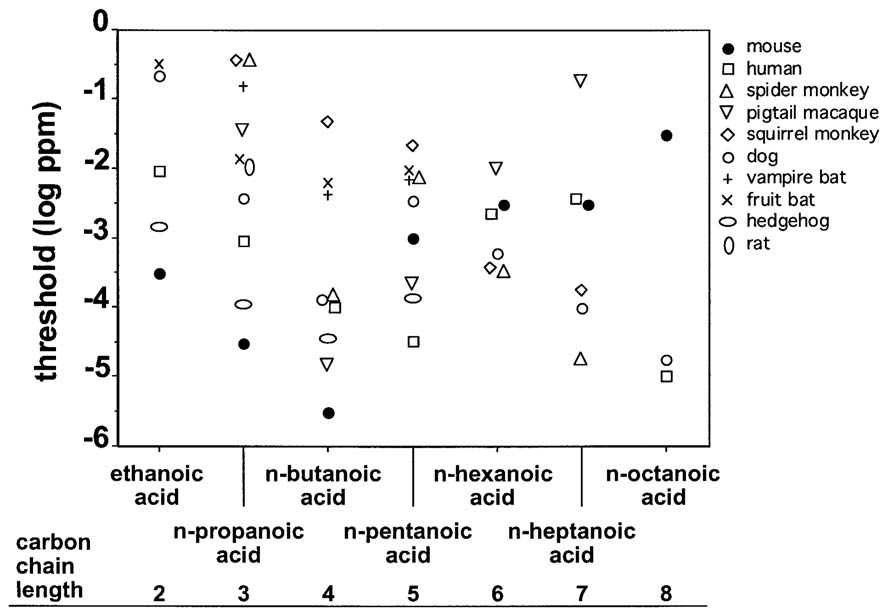
Comparison of the olfactory detection threshold values (expressed as vapor phase concentrations) of the CD-1 mice for aliphatic carboxylic acids and those of other mammalian species. (Human data: [40]; monkey data: [31], [34]; dog, bat, hedgehog and rat data: [41]). Data points of all animal species represent the lowest threshold values of individual animals reported in the literature (and, for mice, in the present study). Data points of the human subjects represent the lowest mean threshold value reported in the literature.

Figure 7 compares the olfactory detection threshold values of the mice for the n-carboxylic acids tested here to those obtained in earlier studies with other mammalian species. Such across-species comparisons should, of course, take into consideration that different methods may lead to widely differing results [20]. However, both the method employed in the present study with CD-1 mice as well as the methods employed in previous studies with other mammalian species were based on instrumental conditioning paradigms which are commonly regarded as the gold standard in animal psychophysics [21]. In this context, it is also interesting to note that the only two studies so far that reported olfactory detection thresholds in mice for one carboxylic acid each correspond favorably with the findings of the present study: Mandairon et al. [22] used a computer-assisted odorized hole-board and reported the olfactory detection threshold of mice (strain C57BL/6J) for n-propanoic acid to be 0.33 ppb which falls exactly into the range of threshold values found in the present study (0.03–1.0 ppb). Similarly, Schmidt [9] used an air-dilution olfactometer and a Y-maze and reported the olfactory detection threshold of mice (strain NMRI) for n-butanoic acid to be 10 ppt which also falls exactly into the range of threshold values found here (3–30 ppt). This suggests that different methods used with the same species do not necessarily lead to differing results. However, it should be noted that Neuhaus [23] reported dramatically lower olfactory detection thresholds for n-carboxylic acids (C_2_ to C_6_, and C_8_) in the dog than the ones depicted in Figure 7. Neuhaus' data were obtained from a single dog using a method and statistics that do not meet today's scientific standards. The replication of Neuhaus' experiments by Moulton et al. [24] using several dogs and a more reliable method and statistics led to the markedly (4–7 orders of magnitude) higher threshold values depicted in Figure 7.

With all these caveats in mind, it seems admissible to state that the CD-1 mice were more sensitive for short-chained (C_2_ to C_4_) aliphatic n-carboxylic acids than the other mammalian species tested so far, but not for longer-chained ones (C_5_ to C_8_). This is remarkable considering that mice have ≈1,060 functional genes coding for olfactory receptors [25] and thus a considerably higher number compared to squirrel monkeys and spider monkeys (≈900) [26] and humans (≈390) [25] which all were more sensitive than the mice with longer-chained carboxylic acids (see Fig. 7). Similarly, mice have a markedly larger relative size of the olfactory bulbs (2.0% of total brain volume) [27] than squirrel monkeys, spider monkeys, and humans (1.2‰, 0.9‰, and 0.09‰ of total brain volume, respectively) [27]. These comparisons lend further support to the notion that genetic features such as the number of functional olfactory receptor genes or neuroanatomical features such as the relative size of the olfactory bulbs are poor predictors of a species' olfactory sensitivity [11], [15].

A comparison of the olfactory detection thresholds obtained in the present study with those obtained in previous studies with other chemical classes shows that the olfactory sensitivity of mice for aliphatic carboxylic acids generally falls into the same range (−1 to −5 log ppm) as that for aliphatic aldehydes [10], aromatic aldehydes [12], alkylpyrazines [11], amino acids [15], and monoterpenes [14]. Interestingly, mice were found to be clearly less sensitive for aliphatic alcohols [28], [29] than for the chemical classes mentioned above. Whether this discrepancy in olfactory sensitivity for different chemical classes is due to differences in their behavioral relevance for the mouse or due to some yet unknown factor remains to be elucidated.

### Odor structure-activity relationships

Our finding of a U-shaped function of olfactory detection thresholds for aliphatic n-carboxylic acids in mice is important as it demonstrates that sensitivity for members of a homologous series of substances is not a simple function of vapor pressure. Corresponding U-shaped functions of olfactory detection thresholds for aliphatic n-carboxylic acids have also been reported in human subjects [30], in pigtail macaques [31], in the hedgehog [32], and in the vampire bat [33], all with a minimum at n-butanoic acid. However, other species such as spider monkeys [31], squirrel monkeys [34], and short-tailed fruit bats [35] were found to display a significant negative (linear) correlation between olfactory detection thresholds and carbon chain length of n-carboxylic acids, and dogs were found to display a non-linear correlation with two minima for members of this chemical class [24]. This suggests that the type of correlation between olfactory detection thresholds and carbon chain length of carboxylic acids may be species-specific.

Using the same method and apparatus as in the present study, CD-1 mice did not show any significant correlation between olfactory detection thresholds and carbon chain length of aliphatic aldehydes [10]. This suggests that the presence and type of correlation between olfactory detection thresholds and carbon chain length of homologous series of substances may not only be species-specific, but also substance class-specific. This notion is also supported by the finding that CD-1 mice display significant negative correlations between discrimination performance and structural similarity of odorants in terms of differences in carbon chain length with acetic esters and 2-ketones, but not with 1-alcohols, n-aldehydes, and n-carboxylic acids [36].

Our finding that branching of the carbon chain had a systematic effect on detectability of carboxylic acids when the branching was next to the functional carboxyl group (see Figure 4) but not when the branching was at the distal end of the carbon chain relative to the functional carboxyl group (see Figure 5) suggests that position of the branching is an important molecular structural feature affecting olfactory sensitivity. This is further supported by our finding that olfactory detection thresholds systematically decreased from 2-methylpentanoic acid over 3-methylpentanoic acid to 4-methylpentanoic acid (see Figure 6). Further studies using other chemical classes of aliphatic compounds and other species are needed to elucidate whether the impact of branching of the carbon backbone of aliphatic substances is also species- and substance class-specific or may be a more generalizable phenomenon.

Functional imaging studies of the rodent olfactory bulb demonstrated that both carbon chain length of aliphatic odorants [37] as well as branching per se and position of branching of the carbon chain [38] led to systematic changes in patterns of glomerular activation. Most of these functional imaging studies employed odor stimuli at the same gas phase concentration – which can be close to an animal's detection threshold for one odorant and way above threshold for another odorant. Given that the neural representations of odorants in the olfactory bulb were also found to change systematically as a function of stimulus concentration [39] it might be a good idea to perform future studies of functional imaging of the olfactory bulb employing concentrations that are matched to a given factor above detection threshold for each odorant instead. This might lead to better across-stimulus comparability of activation patterns and to a better understanding of the neural mechanisms underlying the coding of stimulus intensity. Similarly, employing odorant concentrations that are matched relative to their respective threshold values might help to exclude the possibility of animals using stimulus intensity rather than stimulus quality in studies of discrimination performance. The olfactory detection threshold data presented here may therefore provide useful information for the choice of adequate stimulus concentrations in electrophysiological or imaging studies of the olfactory system or investigations of the discriminative abilities of mice.

## References

[1] Zou Z, Feinstein P, Rivers AL, Mathews GA, Kim A (2004). Postnatal refinement of peripheral olfactory projections.. Science.

[2] Kosaka T, Kosaka K (2005). Intraglomerular dendritic link connected by gap junctions and chemical synapses in the mouse main olfactory bulb: electron microscopic serial section analysis.. Neuroscience.

[3] Souci ER, Albeanu DF, Fantana AL, Murthy VN, Meister M (2009). Precision and diversity in an odor map on the olfactory bulb.. Nat Neurosci.

[4] Mori K, Sakano H (2011). How is the olfactory map formed and interpreted in the mammalian brain?. Ann Rev Neurosci.

[5] Godfrey PA, Malnic B, Buck LB (2004). The mouse olfactory receptor gene family.. Proc Natl Acad Sci USA.

[6] Zhang XH, Zhang XM, Firestein S (2007). Comparative genomics of odorant and pheromone receptor genes in rodents.. Genomics.

[7] Johnson BA, Xu Z, Ali SS, Leon M (2009). Spatial representations of odorants in olfactory bulbs of rats and mice: similarities and differences in chemotopic organization.. J Comp Neurol.

[8] Nara K, Saraiva LR, Ye XL, Buck LB (2011). A large-scale analysis of odor coding in the olfactory epithelium.. J Neurosci.

[9] Schmidt C (1982). Behavioral and neurophysiological studies on the olfactory sensitivity in the albino mouse.. Z Saugetierkd.

[10] Laska M, Joshi D, Shepherd GM (2006). Olfactory sensitivity for aliphatic aldehydes in CD-1 mice.. Behav Brain Res.

[11] Laska M, Persson O, Hernandez Salazar LT (2009). Olfactory sensitivity for alkylpyrazines – a comparative study in CD-1 mice and spider monkeys.. J Exp Zool A.

[12] Larsson L, Laska M (2011). Ultra-high olfactory sensitivity for the human sperm-attractant aromatic aldehyde bourgeonal in CD-1 mice.. Neurosci Res.

[13] Brown RE, Brown RE, MacDonald DW (1985). The rodents. II. Suborder myomorpha.. Social odours in mammals, Vol. 1.

[14] Joshi D, Völkl M, Shepherd GM, Laska M (2006). Olfactory sensitivity for enantiomers and their racemic mixtures – a comparative study in CD-1 mice and spider monkeys.. Chem Senses.

[15] Wallén H, Engström I, Hernandez Salazar LT, Laska M (2012). Olfactory sensitivity for six amino acids: a comparative study in CD-1 mice and spider monkeys.. Amino Acids.

[16] Dykyi J, Svoboda J, Wilhiot RC, Frenkel M, Hall KR (2001). Landolt-Börnstein. Numerical data and functional relationships in science and technology, Group IV, Volume 20, Part C.

[17] Weast RC (1987). Handbook of chemistry and physics, 68th ed.

[18] Bodyak N, Slotnick B (1999). Performance of mice in an automated olfactometer: odor detection, discrimination and odor memory.. Chem Senses.

[19] Doty RL, Laing DG, Doty RL (2003). Psychophysical measurement of human olfactory function including odorant mixture assessment.. Handbook of olfaction and gustation.

[20] Hastings L, Doty RL (2003). Psychophysical evaluation of olfaction in nonhuman animals.. Handbook of olfaction and gustation.

[21] Pearce JM (2008). Animal learning and cognition, 3^rd^ ed.

[22] Mandairon N, Sultan S, Rey N, Kermen F, Moreno M (2009). A computer-assisted odorized hole-board for testing olfactory perception in mice.. J Neurosci Meth.

[23] Neuhaus W (1953). Über die Riechschärfe des Hundes für Fettsäuren.. Z Vergl Physiol.

[24] Moulton DG, Ashton DH, Eayrs JT (1960). Studies in olfactory acuity. 4. Relative detectability of n-aliphatic acids by the dog.. Anim Behav.

[25] Nei M, Niimura Y, Nozawa M (2008). The evolution of animal chemosensory receptor gene repertoires: roles of chance and necessity.. Nat Rev Genet.

[26] Gilad Y, Wiebe V, Przeworski E, Lancet D, Pääbo S (2004). Loss of olfactory receptor genes coincides with the acquisition of full trichromatic vision in primates.. PLoS Biol.

[27] Stephan H, Baron G, Frahm HD, Steklis HD, erwin J (1988). Comparative size of brains and brain components.. Comparative Primate Biology, Vol. 4.

[28] Pho V, Butman ML, Cherry JA (2005). Type 4 phosphodiesterase inhibition impairs detection of low odor concentrations in mice.. Behav Brain Res.

[29] Deiss V, Baudoin C (1997). Hyposmia for butanol and vanillin in mutant staggerer male mice.. Physiol Behav.

[30] Cometto-Muñiz JE, Abraham MH (2010). Structure-activity relationships on the odor detectability of homologous carboxylic acids by humans.. Exp Brain Res.

[31] Laska M, Wieser A, Rivas Bautista RM, Hernandez Salazar LT (2004). Olfactory sensitivity for carboxylic acids in spider monkeys and pigtail macaques.. Chem Senses.

[32] Bretting H (1972). Die Bestimmung der Riechschwellen bei Igeln für einige Fettsäuren.. Z Saugetierkd.

[33] Schmidt U (1975). Vergleichende Riechschwellenbestimmungen bei neotropischen Chiropteren (*Desmodus rotundus*, *Artibeus lituratus*, *Phyllostomus discolor*).. Z Saugetierkd.

[34] Laska M, Seibt A, Weber A (2000). “Microsmatic” primates revisited: olfactory sensitivity in the squirrel monkey.. Chem Senses.

[35] Laska M (1990). Olfactory sensitivity to food odor components in the short-tailed fruit bat, *Carollia perspicillata* (Phyllostomatidae, Chiroptera).. J Comp Physiol A.

[36] Laska M, Rosandher Å, Hommen S (2008). Olfactory discrimination of aliphatic odorants at 1 ppm: too easy for CD-1 mice to show odor structure-activity relationships?. J Comp Physiol A.

[37] Johnson BA, Leon M (2000). Odorant molecular length: one aspect of the olfactory code.. J Comp Neurol.

[38] Johnson BA, Leon M (2007). Chemotopic odorant coding in a mammalian olfactory system.. J Comp Neurol.

[39] Johnson BA, Leon M (2000). Modular representations of odorants in the glomerular layer of the rat olfactory bulb and the effects of stimulus concentration.. J Comp Neurol.

[40] Van Gemert LJ (2003). Compilations of odour threshold values in air, water and other media..

[41] Passe DH, Walker JC (1985). Odor psychophysics in vertebrates.. Neurosci Biobehav Rev.

